# Copy number gain of chromosome 3q is a recurrent event in patients with intraductal papillary mucinous neoplasm (IPMN) associated with disease progression

**DOI:** 10.18632/oncotarget.11501

**Published:** 2016-08-22

**Authors:** Sandra Durante, Silvia Vecchiarelli, Annalisa Astolfi, Elisa Grassi, Riccardo Casadei, Donatella Santini, Riccardo Panzacchi, Claudio Ricci, Salvatore Serravalle, Giuseppe Tarantino, Mirella Falconi, Gabriella Teti, Valentina Indio, Andrea Pession, Francesco Minni, Guido Biasco, Mariacristina Di Marco

**Affiliations:** ^1^ Giorgio Prodi Cancer Research Centre, University of Bologna, Bologna, Italy; ^2^ Department of Experimental, Diagnostic and Specialty Medicine University of Bologna, Sant'Orsola-Malpighi Hospital, Bologna, Italy; ^3^ Department of Medical and Surgical Sciences, University of Bologna, Sant'Orsola-Malpighi Hospital, Bologna, Italy; ^4^ Pathology Unit, Sant'Orsola-Malpighi Hospital, Bologna, Italy; ^5^ Department of Medical and Surgical Sciences, “Lalla Seràgnoli” Hematology-Oncology Unit, University of Bologna, Bologna, Italy; ^6^ DIBINEM—Department of Biomedical and Neuromotor Sciences, University of Bologna, Bologna, Italy

**Keywords:** PDA, IPMN, chromosome 3, NGS, PIK3CA

## Abstract

**Background:**

Intraductal papillary mucinous neoplasm (IPMN) is the most common cystic preneoplastic lesion of pancreatic cancer. We used an approach coupling high resolution cytogenetic analysis (Affymetrix Oncoscan FFPE Array) with clinically-oriented bioinformatic interpretation of data to understand the most relevant alterations of precursor lesions at different stages to identify new diagnostic markers.

**Results:**

We identified multiple copy number alterations, particularly in lesions with severe dysplasia, with 7 IPMN with low-intermediate dysplasia carrying a nearly normal karyotype and 13 IPMN with complex Karyotype (> 4 alterations), showing high grade dysplasia. A specific gain of chromosome arm 3q was found in IPMN with complex Karyotype (92%). This gain of 3q is particularly interesting for the presence of oncogenes such as PIK3CA, GATA2 and TERC that are part of pathways that deregulate cell growth and promote disease progression. Quantitative PCR and FISH analysis confirmed the data. Further demonstration of the overexpression of the PIK3CA gene supports the identification of this alteration as a possible biomarker in the early identification of patients with IPMN at higher risk for disease progression.

**Materials and methods:**

High resolution cytogenetic analysis was performed in 20 formalin fixed paraffin embedded samples of IPMN by Oncoscan FFPE assay. Results were validated by qPCR and FISH analysis.

**Conclusions:**

The identification of these markers at an early stage of disease onset could help to identify patients at risk for cancer progression and new candidates for a more specific targeted therapy.

## INTRODUCTION

Pancreatic Ductal Adenocarcinoma (PDA) is the fourth leading cause of cancer death, and it is projected to overtake breast, prostate, and colorectal cancers to become the second leading cause of cancer-related death by 2030 [1]. To improve the outcome of these patients, in addition to looking for new and more effective therapies, an advancement in early diagnosis may be useful. An increase in the number of patients diagnosed with Intraductal Papillary Mucinous Neoplasm (IPMN) has been recently described, probably due to incidental discovery with new imaging techniques. The prevalence of pancreatic cysts is about 2.5%, a percentage that increases with age: approximately 10% of 70 years old population are diagnosed with pancreatic cysts [2–3]. IPMNs can progress from low to high-grade dysplasia, and finally to invasive adenocarcinoma, but clear data regarding cancer risks are limited. Because of the malignant potential of IPMN, their identification requires imaging analyses and sometimes invasive tests or surgery, but the correct management of these patients is not fully agreed.

The clinical management of these patients is currently based on imaging and cyst fluid analysis. However an aggressive approach could result in over-treating or failure to detect promptly early stages of pancreatic cancer [4–6].

Despite a limited impact on patient care, in the last years the genomic characterization of PDA has advanced substantially [7], showing that PDA is a complex disease consisting of several genetic hits responsible for tumor onset, growth and maintenance. In particular, the stromal microenvironment was found to be the main dynamic compartment, enabling tumor growth and progression [8] as well as pancreatic stem cells whose role in carcinogenesis has already been established. Although the parental clones accumulate additional mutations during tumor progression and metastasis to distant organs, we can narrow the attention on twelve principal signaling pathways involved in PDA development. However, to explain the great complexity of PDA, it must be emphasized that not all tumors show alterations in all of these pathways, and key mutations appear to differ from one cancer to another [9–10]. Some studies, performed on PDA and precursor lesions, as intraepithelial neoplasm (PanIn) [11–12] and IPMN [13–15] strengthened the notion that metastatic subclones are pre-existent within primary carcinoma or precursor lesions, both in terms of founder mutations and ofrearrangements [16–17].

Genetic studies support the hypothesis that PanINs can be a precursor to invasive pancreatic cancer, and have shown that the increasing morphologic grades of dysplasia in PanIN are accompanied by the accumulation of genetic alterations. These genetic alterations appear to occur after telomere shortening and KRAS gene mutations, as they are usually not found in low- grade PanINs, but instead are found in higher-grade PanIN lesions. Some of the genetic changes in PanINs appear to be associated with progression [18].

However, to date, despite the discovery of numerous genetic markers and related signaling pathways, we have not yet identified a specific marker that is able to detect early stages of disease already primed for carcinogenesis, and to guide towards an effective targeted therapy that could improve the quality of life of the patients. There are only few studies that discovered the alterations already present in precursor lesions, that can be associated with a worse clinical course of the disease. Most studies showed the very high frequency of KRAS and GNAS mutations in IPMN, associated with mutations of TP53 in a smaller subset of more aggressive lesions, and by some recurrent aneuploidies and focal deletions of tumor suppressor genes [19–23].

In this study we performed a high resolution cytogenetic analysis of both early and advanced IPMN lesions to correlate genomic profiling with histological features and disease progression. The aim of this study is to identify the genetic aberrations that are suggestive of a precursor lesion at higher risk of evolving into fully blown PDA, thus potentially providing the physicians with informative data that can aid clinical decision-making.

## RESULTS

### Clinicopathological data

**Figure 1:**
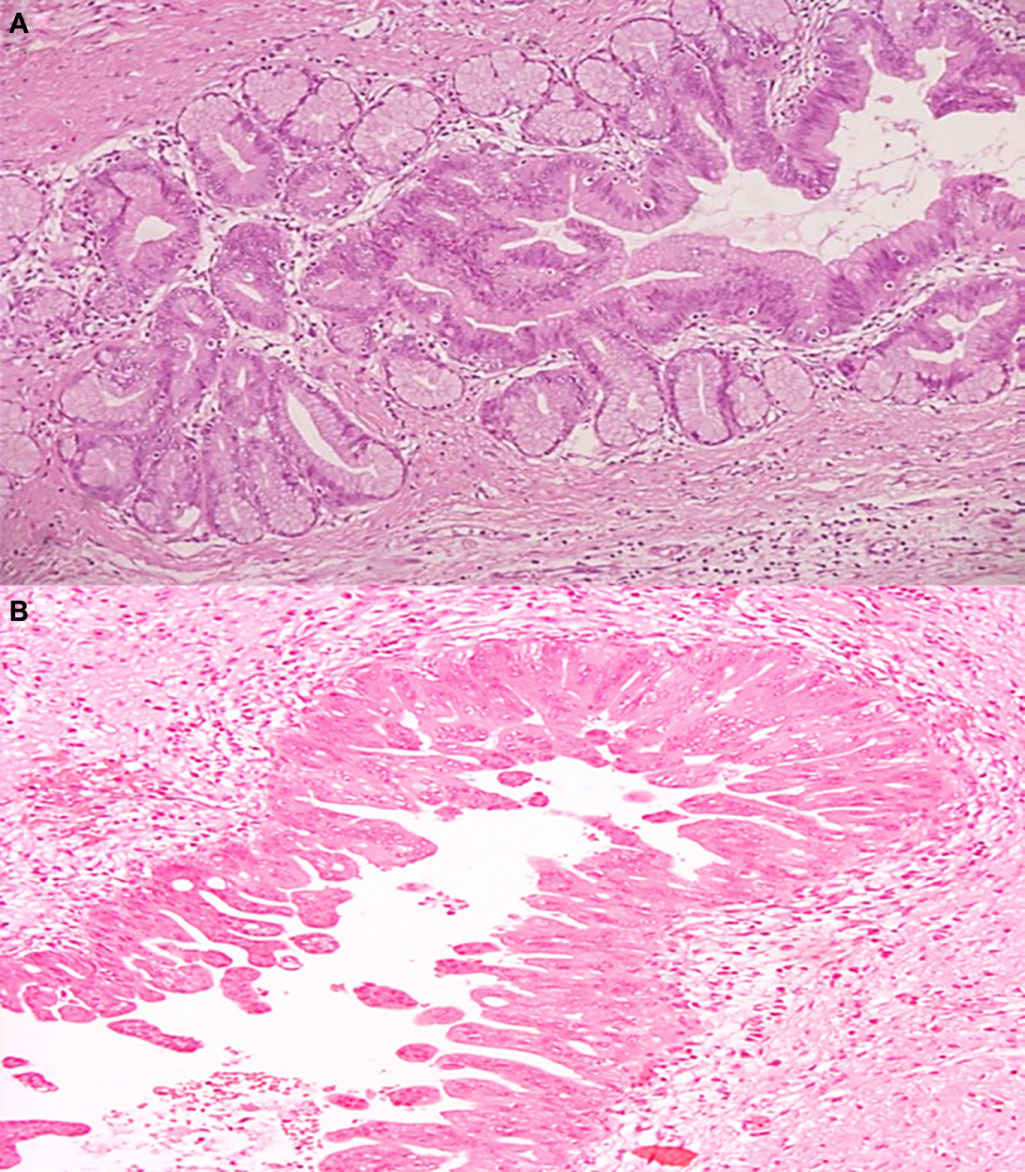
(**A**–**B**) Hematoxylin and eosin stained sections of IPMN (A) Microscopic finding of the resected specimen demonstrates pancreatic branch duct with low-intermediate-grade gastric type IPMN (H&E ×20). (B) Microscopic finding of the resected specimen demonstrates pancreatic main duct with high-grade pancreatobiliary-type IPMN (H&E ×20).

A total of 20 surgical specimens of IPMN were collected and classified according to WHO 2010 classification. Of the 20 IPMNs, nine (45%) were multifocal, seven (35%) were in the pancreatic head and four (20%) involved pancreatic tail. Macroscopically 14 samples (70%) were mixed-type, while 4 (20%) branch-duct type and 2 (10%) main-duct type. Dysplasia was low-intermediate in 7 cases, while high grade dysplasia was found in 13 out of 20 samples (Figure 1A–1B). In 4 IPMNs (20%) micro-invasive carcinoma areas were identified (< 1 mm), while two (10%) were associated with an invasive carcinoma (Table 1).

**Table 1:** Summary of IPMN patients' data, grading according to WHO 2010

Demographic and histopathological data	
Age – years	
Median	71
Range	55–84
Sex – no. (%)	
Female	9 (45)
Male	11 (55)
Site – no (%)	
Head/Uncinate process	7 (35)
Head/Body	3 (15)
Body/Tail	2 (10)
Tail	4 (20)
Diffuse	4 (20)
Type-no (%)	
Main duct	2 (10)
Branch duct	4 (20)
Mixed type	14 (70)
Histotype – no (%)	
Pancreaticobiliary	6 (30)
Gastric	2 (10)
Gastric/pancreaticobiliary	10 (50)
Intestinal	1 (5)
Oncocytic	1 (5)
Lesion – no (%)	
Low-grade	3 (15)
Intermediate-grade	4 (20)
High-grade	13 (65)
Associated carcinoma – no(%)	
Invasive	2 (10)
Microinvasive(< 1 mm)	4 (20)

Median follow-up was 34,0 ± 7,4 months. Of 20 cases analyzed, 2 patients died: 1 case for an independent cause (peritoneal recurrence of previous gastric cancer) and 1 for pancreatic cancer developed in the pancreatic residual.

### Mutation analysis

Oncogenic mutations in KRAS, GNAS and TP53 were detected by Sanger sequencing (Table 2). 6/20 IPMN showed a mutation in KRAS (G12R, G12V or G12D), 2/20 carried mutations at codon 201 of GNAS and 5/20 showed a mutation in TP53. No significant association with respect to the grade of dysplasia was found for KRAS, GNAS or TP53 mutations, probably due to the small sample size. However it is noticeable that GNAS mutations were found only in 2/7 low grade dysplasia samples, while the 5 patients carrying TP53 mutations all displayed high grade dysplasia. Among patients with TP53 mutation, one patient experimented pancreatic cancer onset from the surgical residual and died for that cause.

**Table 2:** Association between the grade of dysplasia, the number of copy number gains and losses and the mutational status of KRAS, TP53 and GNAS

Sample	Dysplasia	Gains	Losses	Mutations
Onco009	Intermediate-grade	0	0	
Onco013	Intermediate-grade	0	0	
Onco014	Low-grade	0	0	
Onco004	Low-grade	1	0	
Onco010	Intermediate-grade	1	0	KRAS G12D
Onco011	Intermediate-grade	1	0	GNAS R201C
Onco012	Low-grade	1	0	KRAS G12V / GNAS R201H
Onco025	High-grade	5	14	KRAS G12R
Onco015	High-grade	5	2	TP53 G245S
Onco026	High-grade	4	0	TP53 G245S
Onco024	High-grade	5	0	
Onco017	High-grade	7	3	
Onco008	High-grade	5	0	
Onco020	High-grade	6	0	
Onco019	High-grade	7	10	KRAS G12V / TP53 G245S
Onco016	High-grade	10	1	TP53 R249S
Onco021	High-grade	5	1	
Onco022	High-grade	8	10	TP53 G245S
Onco018	High-grade	6	1	KRAS G12D
Onco023	High-grade	17	0	KRAS G12V

### High resolution cytogenetic profile

**Figure 2:**
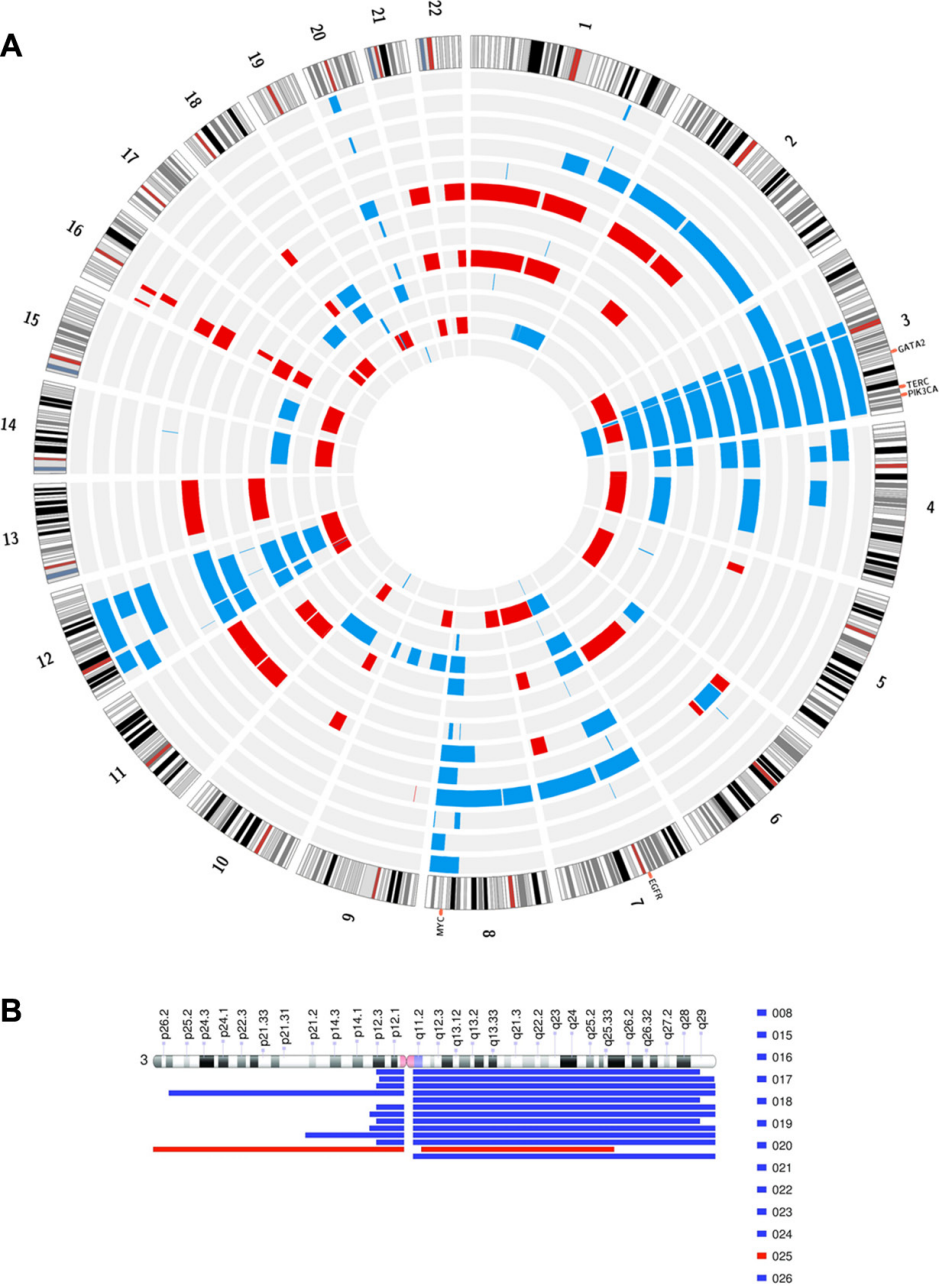
(**A**) Illustration of circular plot for regions of statistically significant copy number alterations in the IPMN with complex karyotype. The lines indicate concordance (frequency among the sample group gain/loss). Red and blue lines indicate loss and gain, respectively. (**B**) Representation of regions of copy number alteration of chr 3 in IPMN samples with complex karyotype.

20 samples of IPMN were analyzed by genome-wide copy number assay using the Oncoscan FFPE system. Multiple copy number alterations were identified, thus defining two major categories of IPMN at the genomic level: 7 IPMN were characterized by a nearly normal karyotype, with no copy number alteration or at most only one focal gain (Table 2), while 13 IPMN carried a complex karyotype, with more than 4 macroscopic copy number gains or losses, and 10 copy number alterations each, on average (Figure 2A). Karyotype status correlated strongly with histological grade, since all the 13 cases with high-grade dysplasia were carriers of a complex karyotype, compared to the 7 normal karyotype that showed only low grade/intermediate dysplasia (*p* < 0.0001, Fisher exact test).

A recurrent and specific gain of chromosome arm 3q was detected in 12 out of 13 (92%) of IPMN with complex Karyotype (Figure 2B), encompassing known oncogenes classified in Cancer Gene Census, such as PIK3CA, GATA2 and TERC, thus possibly linked to the deregulation of cell growth and to the progression of disease. This specific alteration was the one most significantly associated with the grade of dysplasia (*p* = 0.0001), while no association was found between the presence of 3q gain and any specific IPMN histotype (gastric, intestinal, pancreatobiliary or oncocytic types). Other recurrent gains involved chr 8q, chr 12, chr 7, while frequent losses involved chr 16, chr 21 and chr 22. Putative target genes involved in chromosome arm loss are PALB2 on chr 16, and SMARCB1, CHEK2, NF2 and EP300 on chromosome 22.

**Figure 3:**
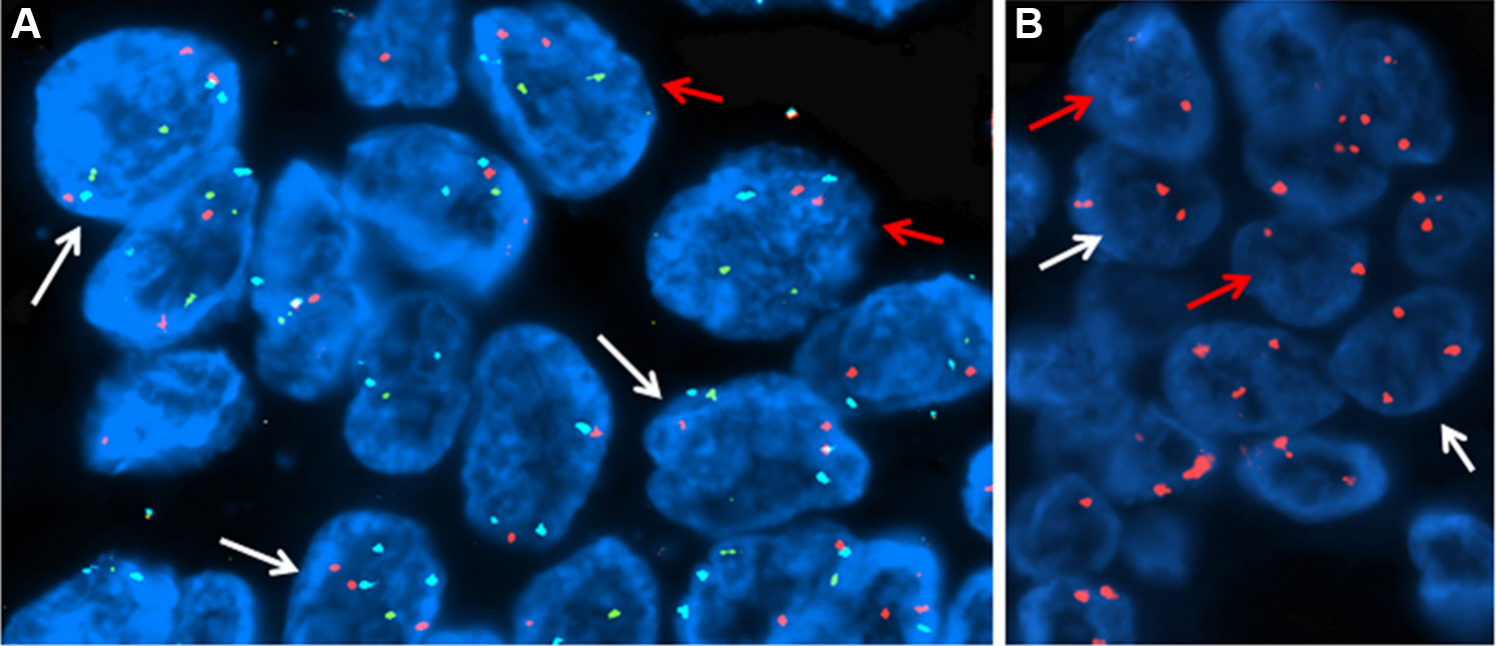
FISH analyses (**A**) Copy number gain of MYC gene on chr 8: about the 50% of the cells showed three signals for MYC gene (orange) and centromere of chromosome 8 (aqua) but normal signals for the IGH gene (green), suggesting a gain of chr 8. (**B**) Detection of chr 3 gain: about 60% of cells were abnormal showing three orange signals for the centromere of chromosome 3, suggesting a gain of chr3. In both panels, the red arrows indicate normal cells while the white arrows point at the abnormal ones.

In the IPMN with complex karyotype a second recurrent finding was the gain of chromosome arm 8q (45%) (Supplementary Figure S1) where the MYC oncogene resides. In particular in one case, the region was focally amplified (Supplementary Figure S2). We employed FISH analysis to validate copy number changes on chromosome 8, confirming copy number gain observed in Oncoscan analysis (Figure 3A)

Interestingly in nearly half of the patients with high grade dysplasia we also observed additional gain in chromosome 7p11, where EGFR gene is located (Supplementary Figure S3), and gain of chr 12, where MDM2 and KRAS are located (40%).

### 3q arm gain is a recurrent event in complex karyotype IPMN

**Figure 4:**
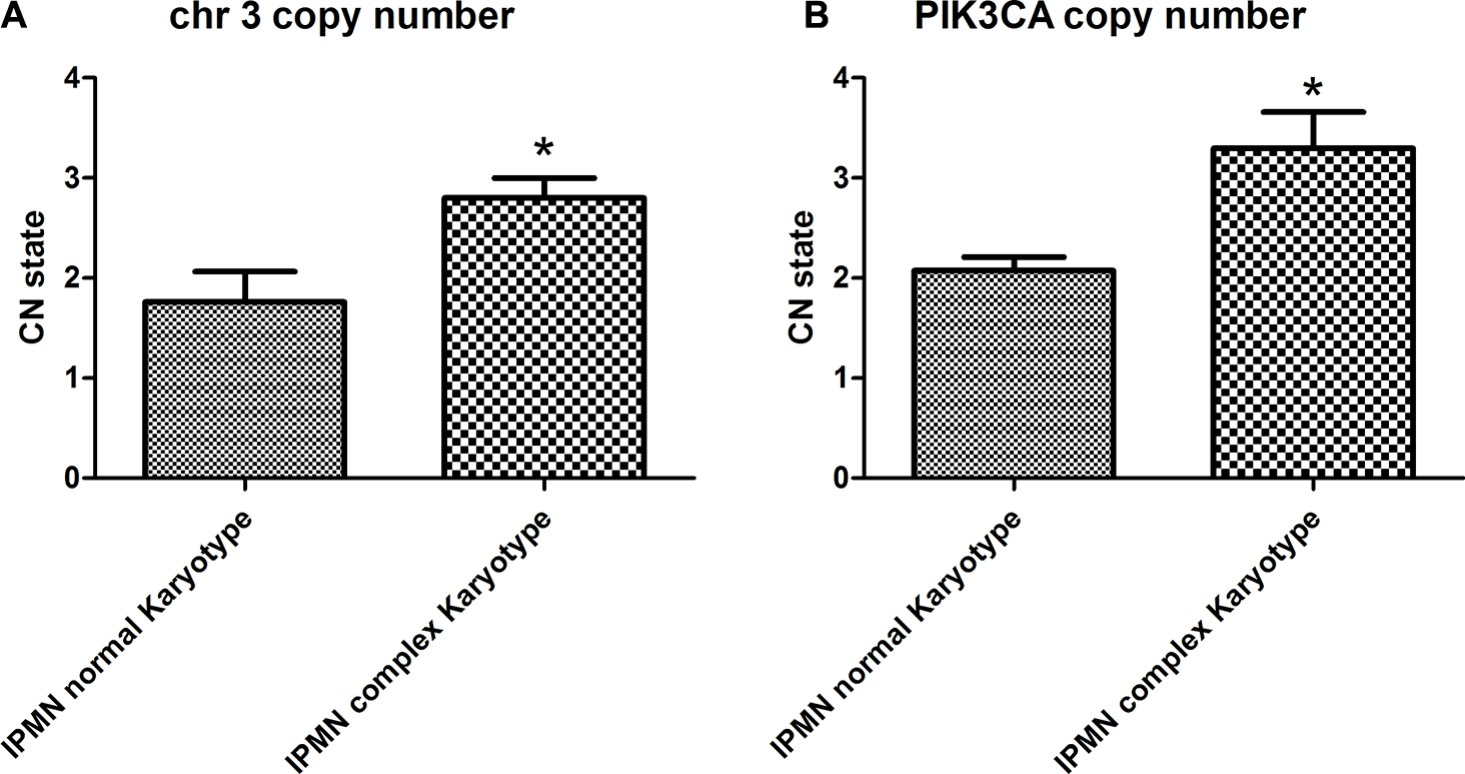
Copy number quantification of chromosome 3 by qPCR (**A**) Chromosome 3 copy number state in IPMN with complex karyotype compared to normal karyotype. The difference is statistically significant, with a *p-value* < 0.008. (**B**) Focusing specifically on PIK3CA gene, qPCR confirmed the gain of this genomic region in IPMN with complex karyotype with a *p-value* < 0.004.

**Figure 5:**
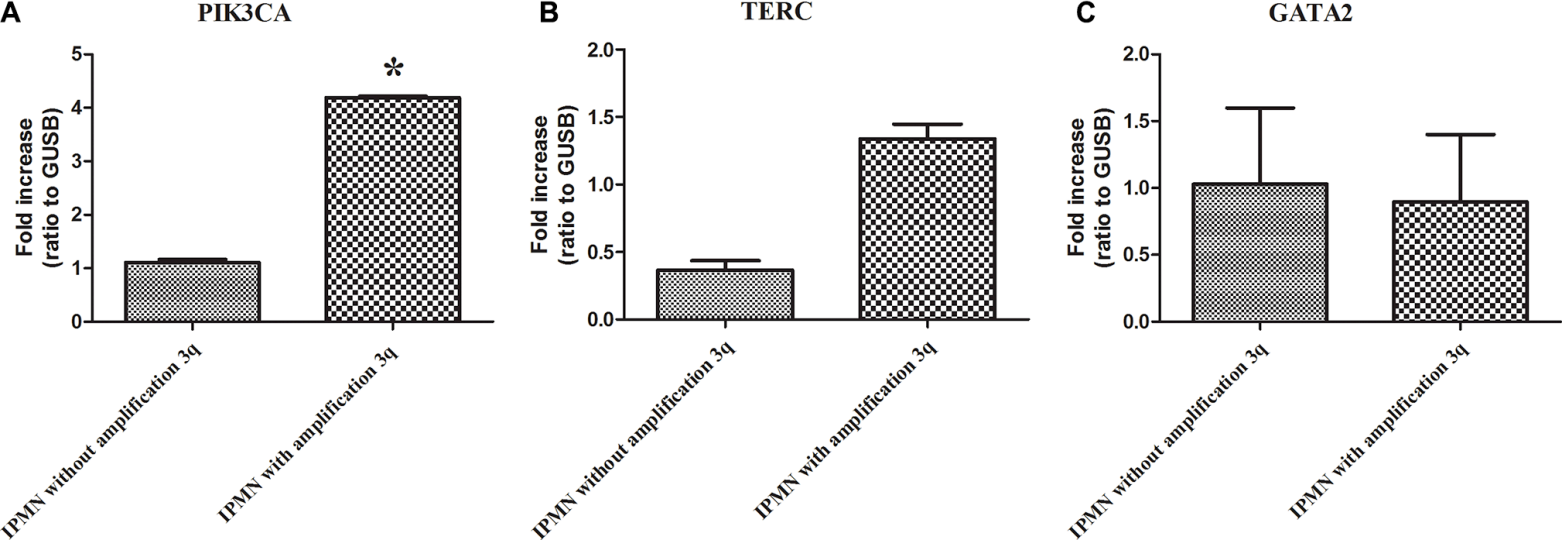
(**A**) Expression of PIK3CA mRNA in IPMN without gain of 3q compared to IPMN with 3q gain. The difference is statistically significant (*p* < 0.0004). (**B**) Expression of TERC mRNA in IPMN without gain of 3q compared to IPMN with 3q gain. (*p* < 0.01). (**C**) Expression of GATA2 mRNA in IPMN without gain of 3q compared to IPMN with 3q gain. Each individual assay was performed in triplicate and expressed as mean ± SD; * represents a very significant difference of IPMN without gain of 3q compared to IPMN with gain of 3q.

The gain of chromosome 3 was confirmed both by FISH and quantitative qPCR. FISH analysis confirmed the presence of a gain of chr 3 in patients with IPMN with complex Karyotype, supporting the data obtained by whole genome assay (Figure 3B). qPCR with primers located in 3p12.3 confirmed a significant gain of chr 3 in IPMN with complex karyotype with a *p-value* of 0.008, compared with IPMN with normal karyotype (Figure 4A). Focusing specifically on PIK3CA gene, qPCR confirmed the gain of this genomic region in IPMN with complex karyotype (Figure 4B). The mRNA expression of PIK3CA, GATA2 and TERC, the candidate oncogenes on chr 3q, were evaluated by qRT-PCR in IPMN with normal karyotype and IPMN with gain of 3q, and the results were compared to normal pancreatic tissue (Figure 5). Results showed a 4-fold up regulation of PIK3CA in IPMN with gain of 3q compared to IPMN with normal chr3, with a *p* value < 0.0004 (Figure 5A). TERC expression was 2-fold upregulated in IPMN with chr 3q gain, with *p* value < 0.01 (Figure 5B), while expression of GATA2 showed no difference between the two groups (Figure 5C).

## DISCUSSION

The poor outcome of patients with PDA is due to the accumulation of multiple genetic alterations, to the contribution of the stromal microenvironment to chemo and radio-resistance and to the delay in clinical diagnosis. Moreover, the identification and targeting of molecular alterations is impaired by the rich stromal microenvironment that overtakes the tumor cellularity and by the high number of genetic lesions that are accumulated during the transition from pre-neoplastic conditions to fully malignant phenotype. Intraductal Papillary Mucinous Neoplasm (IPMN) are precursor lesions that represent a great opportunity for early detection since they can be managed surgically time before the full acquisition of malignant features. This can indeed be done only if we identify morphological or molecular features that can predict the evolution of these lesions towards invasive carcinoma. There are some molecular events that have been proposed as markers of a more aggressive phenotype of IPMN, such as inactivation of p53 that was found in IPMNs with high grade dysplasia [22]. Moreover, it has been demonstrated that p53 overexpression is strongly associated with invasive phenotype in IPMN and can be considered a biomarker of malignant and aggressive behaviour [23]. Indeed in our series we showed that TP53 mutation occurs only in high grade dysplasia, even if the association is not statistically significant, probably due to small sample size. Many reports showed the very high frequency of mutations of KRAS in IPMN, ranging from 50 to 70% of samples [19–20]. Also in our series KRAS was the most frequently mutated gene, even if the overall proportion of mutated samples was lower (30%).

Here we used an approach with high resolution cytogenetic analysis of both early and advanced IPMN lesions to correlate genomic profiling with histological features and disease progression. 20 samples of IPMN were analyzed by genome-wide copy number assay using the Oncoscan FFPE system, and multiple copy number alterations were identified, thus defining two major categories of IPMN at the genomic level: 7 IPMN were characterized by a nearly normal karyotype, with none or one focal alteration at most, while 13 IPMN carried a complex karyotype, with more than 4 alterations each. The average number of alterations in complex karyotype cases was 10 for each patient, with gains outnumbering losses by a factor 2. This suggests that there are genetic differences even within IPMN depending on the degree of dysplasia. In nearly half of the patients with high grade dysplasia we also observed a gain in chromosome 7p11, where the EGFR gene is located and the gain of chromosome arm 8q where the MYC oncogene resides.

The scientific literature reports that the amplification of the 8q24 locus involves the MYC oncogene, whose amplification is significantly associated with poor outcome in the adenosquamous subtype of pancreatic carcinoma [24] and in cervical dysplasia [25].

However our attention was focused on the 3q arm gain since it was clearly associated with IPMN with complex karyotype and high-grade dysplasia. This region is particularly interesting because it includes oncogenes as PIK3CA, GATA2 and TERC thus possibly linked to the deregulation of cell growth and to the progression of disease. In particular, PIK3CA and TERC appear to be the most relevant target genes of chr 3q gain, since the mRNA expression of PIK3CA and TERC were upregulated in IPMN with gain of 3q compared to IPMN with normal karyotype. These data on the gain and overexpression of PIK3CA and TERC suggests that they can be evaluated further as useful biomarkers in the early identification of patients with high risk IPMN. PI3K signaling is often deregulated in tumors and contributes to the oncogenic process. It is known that PI3K pharmacological blockade significantly reduces the proliferation rate in culture, suggesting that inhibition of PI3K might prove beneficial in experimental therapies [26] especially in IPMN patients with overexpression of PIK3CA.

It is possible that the increased copy number of TERC, encoding one telomerase component, has a pro-oncogenic effect in consideration of the ability of telomerase to prevent telomere shortening and allow cells to escape from senescence [27].

TERC and c-myc copy number gains are associated with the progression of the disease [24]. These data suggest a role for MYC in the initiation and progression of the preneoplastic stages of this aggressive disease. The management of patients with IPMN lesions would require the identification of markers of a more aggressive disease to aid clinical decision-making, and to suggest a therapeutic strategy based on tailored surgical resection possibly combined with a specific target therapy, where PIK3CA involvement may have a significant clinical impact.

To detect the overexpression of these markers at an early stage of cancer initiation would help to improve the management of the high risk category of these patients, and possibly to identify new candidates for a more specific targeted therapy.

## MATERIALS AND METHODS

### Sample collection

A collection of 20 FFPE tissue samples of IPMN surgically resected between 2010 and 2014 was obtained from the archives of the Department of Pathology at the Sant'Orsola Malpighi Hospital of Bologna. Patients' characteristics are detailed in Table 1. The current study was conducted according to the principles of the Declaration of Helsinki, and written informed consent was obtained from all participants. The study was previously approved by the Independent Ethics Committee of Sant'OrsolaMalpighi Hospital (Bologna, Italy). For all the patient included in the study a 2-years follow-up was available. Tumor samples were subjected to routine histopathological examination by expert pathologists. Sections were cut and stained with hematoxylin and eosin, and samples were observed under light microscope using an Eclipse E800 Nikon (Nikon, Tokyo, Japan). IPMN were classified as low, intermediate or high grade dysplasia based on cytoarchitectural atypia. IPMN were classified, according to epithelial morphology, in gastric, oncocytic, pancreatobiliary or intestinal type, and depending on duct involvement in main-duct type, branch-duct type and mixed type. The presence of invasive cancer or micro invasive cancer was also described.

### DNA extraction

DNA was extracted from tissues after enrichment for the neoplastic component, using manual macrodissection. DNA was extracted using QIAamp DNA micro Kit (QIAGEN) following the manufacturer's recommended protocol and according to the Standard Operating Procedure of the laboratory. DNA was quantified using the Quant-IT PicoGreen dsDNA Assay Kit (Thermo Fisher, Monza, Italy) against a reference standard curve. The concentration of the DNA stock was adjusted to 12 ng/μl using reduced EDTA TE buffer (10 mM Tris-HCl, 0.1 mM disodium EDTA, pH 8).

### Sanger sequencing

Mutations were analyzed by Sanger sequencing with specifically designed primer on exons carrying hotspot mutations of KRAS and GNAS, and on all the coding exons of TP53. PCR reactions were performed using the AmpliTaq Gold 360 Master Mix (Life Technologies, USA) or FastStart TAQ DNA polymerase (Roche, Milan, Italy) and visualized by agarose gel electrophoresis. Subsequently, amplicons were purified and sequenced using the BigDye Terminator reaction mix v1.1, and analyzed on the 3730 Sequence Analyzer (Applied Biosystems). All reactions were performed in duplicate, including the DNA extraction process.

### Oncoscan assay

The DNA extracted from 20 FFPE a concentration of 12 ng/ul were processed with the OncoScan FFPE Express 2.0 System (Affymetrix, Santa Clara, CA). Data analysis was performed using the Oncoscan Nexus Express Software (Biodiscovery, Hawthorne, CA) and whole chromosome gains and losses and copy number aberrations (deletions and duplications) were determined. In order to remove false positive alterations we have performed a downstream screening using as filtering parameter the percentage of overlapping with physiological CNV listed in the Database of Genomic Variants (DGV-release of July 2015). Also, since the application of this technique to FFPE-derived DNA carries an intrinsic higher background noise that inflates the number of false positive calls particularly in small intra-genic regions, this parameter was rated in relation to the length of the fragments. Chromosomal gains and losses were visualized by Circo Plot (http://circos.ca/). Identification of oncogenes as putative targets of copy number alterations was performed through data integration with the Cancer Gene Census database (http://cancer.sanger.ac.uk/cosmic/census).

### Copy number quantification

Copy number quantification of chromosome 3 gain and specifically of the PIK3CA genomic region was carried out using quantitative real-time PCR(qPCR) on the Light Cycler 480 Real Time PCR System (Roche, Penzberg, Germany). qPCR assay was performed with TaqMan Genotyping Master Mix (Thermo Fisher) and 500 nM of primer TaqMan^®^Copy Number Assays Robo (Hs02653866) for target chr3 and PIK3CA (Hs02608866_cn) for the specific region of 3q. Taqman Copy Number Reference human, RNase P assay was used as the endogenous reference gene.

### FISH

Fluorescence *In Situ* Hybridization (FISH) was performed on paraffin-embedded IPMN tissue pretreated using the Paraffin Pretreatment Reagent Kit (Abbott Molecular Inc., Germany) following the manufacturer's protocol. Two Abbott Molecular's Vysis probes (Abbott Molecular Inc., Germany) for chromosome 3 and for MYC gene and chromosome 8 were employed according to manufacturer's instructions. Specifically, the Vysis CEP 3 (D3Z1) SpectrumOrange Probe was used to probe the 3p11.1-q11.1 Alpha Satellite DNA (orange), while the IGH/MYC/CEP 8 probe identified the IGH gene on chr14q32 (green), the MYC gene on chr8q24 (orange) and the centromeric 8p11.1-q11.1 (aqua). Haematoxylin-eosin (H&E) stained sections (4 mm) were cut before FISH sections (4 mm), to confirm tumor presence.

### Quantitative real time polymerase chain reaction (qRT-PCR)

Total RNA was extracted by RecoverAll Total Nucleic Acid Isolation Kit (Ambion ThermoFisher, Monza, Italy) quantified using a NanoDrop^®^ ND- 1000 UV-Vis Spectrophotometer (Thermo Scientific, Wilmington, DE, USA) and cDNA was transcribed with reverse transcriptase SuperscriptIII (Invitrogen, Carlsbad, CA, USA). mRNA expression was analyzed by quantitative Real Time PCR using Light Cycler 480 Real Time PCR System (Roche). For the analysis the following TaqMan assays (Thermo Fisher Scientific Inc.) were used: PIK3CA (Hs00907957_m1), GATA2 (Hs00231119_m1) and TERC (Hs03454202_s1). The relative gene expressions were normalized to GUSB housekeeping gene (Hs00939627_m1) and the resulting data were expressed as fold change using the ΔΔCT method, as recommended by the manufacturer (User Bulletin No.2 P/N 4303859, Applied Biosystems). Data were expressed as the average ± SEM and were representative of three independent experiments.

### Statistical analysis

The Fisher exact test was performed to evaluate associations between segmental chromosomal rearrangements and clinical features. *p* < 0.05 was considered statistically significant. Quantitative data were compared by Mann-Whitney *U* test.

## SUPPLEMENTARY MATERIALS FIGURES


