# Supplementary material for: Copy number gain of chromosome 3q is a recurrent event in patients with intraductal papillary mucinous neoplasm (IPMN) associated with disease progression
Source: Oncotarget. 2016 Aug 22;7(46):74797–806. doi: 10.18632/oncotarget.11501 (PMC5342702; doi:10.18632/oncotarget.11501)
Supplement: Supplementary file 1 [file oncotarget-07-74797-s001.pdf]

Copy number gain of chromosome 3q is a recurrent event in patients with intraductal papillary mucinous neoplasm (IPMN) associated with disease progression

Supplementary Materials

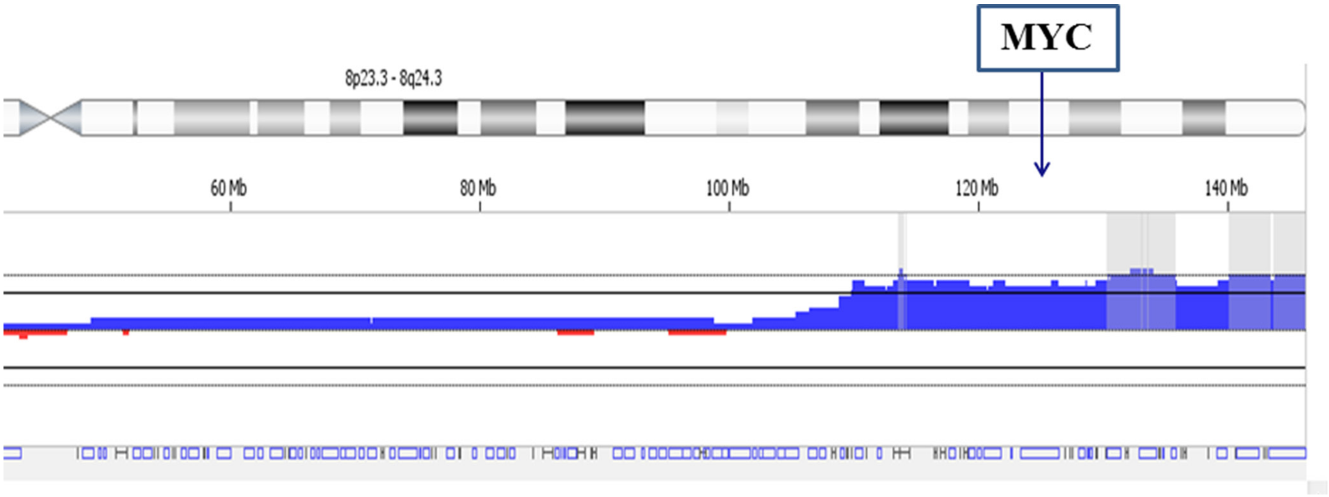

Supplementary Figure S1: Gain of 8q in IPMN with complex karyotype. In particular we can observe the gain of MYC locus in 45% of samples.

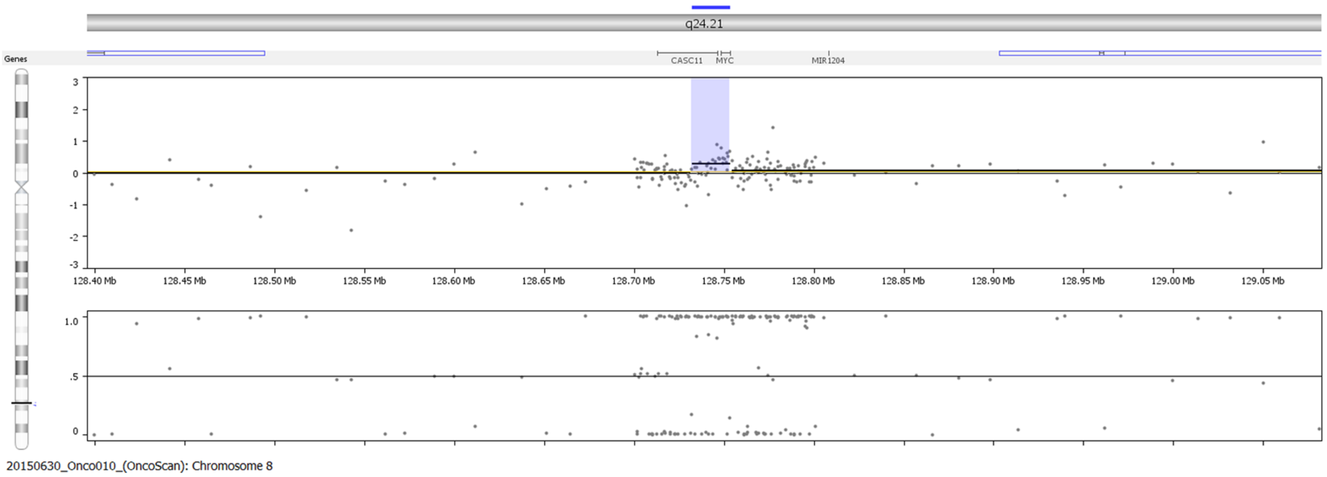

Supplementary Figure S2: A specific sample where the gain of MYC gene is focal.

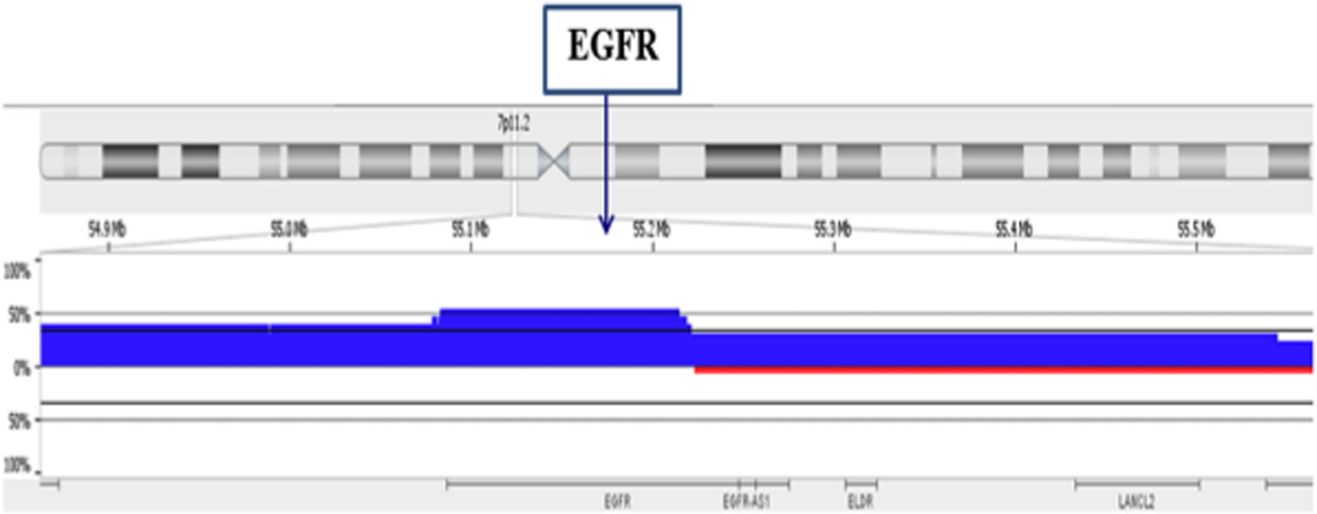

Supplementary Figure S3: Gain of 7p in IPMN with complex karyotype. In particular we can observe the gain of EGFR locus in 45% of samples.
